# Knowledge, attitude and practices of COVID-19 among medical laboratory professionals in Zambia

**DOI:** 10.4102/ajlm.v10i1.1403

**Published:** 2021-03-04

**Authors:** Adon Chawe, Ruth L. Mfune, Paul M. Syapiila, Sharon D. Zimba, Pipina A. Vlahakis, Samson Mwale, Kapambwe Mwape, Memory Chirambo-Kalolekesha, Misheck Chileshe, Joseph Mutale, Tobela Mudenda, Grace Manda, Victor Daka

**Affiliations:** 1 Laboratory Department, St. Francis Mission Hospital, Katete, Zambia; 2 Department of Clinical Sciences, Faculty of Medicine, Michael Chilufya School of Medicine, Copperbelt University, Ndola, Zambia; 3 Department of Clinical Sciences, Faculty of Medicine, Chikankata College of Biomedical Sciences, Chikankata, Zambia; 4 Department of Basic Science, Faculty of Medicine, Michael Chilufya School of Medicine, Copperbelt University, Ndola, Zambia; 5 Department of Biomedical Sciences, Tropical Diseases Research Centre, Ndola, Zambia; 6 Laboratory Department, Mary Begg Health Services, Ndola, Zambia; 7 Laboratory Department, Kabompo District Hospital, Kabompo, Zambia; 8 Department of Pathology, Ndola Teaching Hospital, Ndola, Zambia; 9 Laboratory Department, Kalomo District Hospital, Kalomo, Zambia

**Keywords:** COVID-19, medical laboratory professional, knowledge, attitude, practices

## Abstract

**Background:**

Coronavirus disease 2019 (COVID-19) is a novel disease that has spread to nearly every country worldwide. Medical laboratory professionals are key in the fight against COVID-19 as they provide confirmatory diagnosis for subsequent management and mitigation of the disease.

**Objective:**

This study investigated the knowledge, attitude and practices of COVID-19 and their predictors among medical laboratory personnel in Zambia.

**Methods:**

We conducted a cross-sectional study among medical laboratory professionals in Zambia from 10 to 29 June 2020. Data were collected using Google Forms and exported to Statistical Package for Social Sciences version 23 for statistical analysis. Independent predictors of COVID-19 knowledge and practices were determined. Adjusted odds ratios (AOR) and their 95% confidence intervals (CI) are reported.

**Results:**

A total of 208 medical laboratory professionals, 58.2% male, participated in the study. The majority of respondents had good knowledge (84.1%) and practice (75.0%) regarding COVID-19. Predictors of good knowledge included having a bachelor’s degree (AOR: 5.0, CI: 1.13–22.19) and having prior COVID-19 related training (AOR: 8.83, CI: 2.03–38.44). Predictors of good practice included having a master’s or Doctor of Philosophy (PhD) qualification (AOR: 5.23, CI: 1.15–23.87) and having prior COVID-19 related training (AOR: 14.01, CI: 6.47–30.36).

**Conclusion:**

Our findings revealed that medical laboratory professionals in Zambia have good knowledge regarding COVID-19. There is need for continuous professional development to ensure that medical laboratory professionals are well informed and aware of best practices to aid in curbing the pandemic.

## Introduction

Coronavirus disease 2019 (COVID-19) is a respiratory disease caused by severe acute respiratory syndrome coronavirus 2.^1^ Coronaviruses have been known to affect humans, infecting the respiratory tract and causing infections ranging from mild to severe.^2^ In the past, different strains of coronaviruses have caused severe acute respiratory syndrome and Middle East respiratory syndrome. Research has shown that COVID-19 is more contagious compared to the previous outbreaks, but less lethal.^3^ Transmission occurs through the inhalation of droplets or contact with surfaces that have been contaminated with the severe acute respiratory syndrome coronavirus 2. As of this writing, there is currently no known vaccine or documented specific treatment for COVID-19 disease.^4^ Drugs that show potential to treat critically ill patients are still being investigated for safety and efficacy.^4,5,6^ Prevention and control of the spread of COVID-19 is done by social distancing, wearing face masks to prevent both the inhalation and transmission of infectious droplets, as well as effective hand hygiene by regularly washing hands or using alcohol-based hand sanitisers.^7,8^

The first cases of COVID-19 were reported in December 2019 in Wuhan, Hubei province, central China. Since then, the disease has spread to nearly every country worldwide leading to the World Health Organization declaring COVID-19 a pandemic on 11 March 2020.^9^ As of 27 October 2020 there were 44 055 642 confirmed cases and 1 168 306 deaths reported globally, with 1 740 720 confirmed cases and 38 830 deaths reported in Africa.^10^ In Zambia, 16 243 confirmed cases and 348 deaths were reported as of 27 October 2020, including a high proportion of community deaths.^11,12^

Few publications and national situation reports exist that detail the number of healthcare workers (HCWs) including medical laboratory personnel around the world infected with COVID-19.^13^ In China where the disease first started, 2055 HCWs were infected as of 24 March 2020.^14^ In the United States, 9282 HCWs were infected as of 14 April 2020.^15^ The World Health Organization estimates that over 1000 HCWs in Africa had been infected with COVID-19 by 23 July 2020.^16^ Information on the source of infection among HCWs in countries that have recorded the disease remains limited.

Medical laboratory professionals are key personnel in the diagnosis of COVID-19 in Zambia. Although they are not in the front line, which precludes them from prominence, their role in providing confirmatory diagnosis is the main basis upon which cases are identified and clinical management is instituted. The work areas of biomedical laboratory professionals are very hazardous due to both suspected and unsuspected infectious agents. Lack of knowledge and good attitude, as well as poor laboratory practices, can have a twofold effect. Firstly, a wrong diagnosis leading to wrong patient management could portend severe consequences for the patient as well as undermine transmission prevention efforts; this is particularly true with COVID-19. Secondly, poor attitude and practices could result in safety incidents (such as infection transmission) which could be deleterious to both the concerned staff and their immediate environment, including co-workers, families and patients or laboratory clients.^17^

Therefore, this study aimed at evaluating the knowledge, attitudes and practices of medical laboratory professionals in Zambia who are integral in the diagnosis of COVID-19.

## Methods

### Ethical considerations

Ethical clearance for this study was obtained from the Tropical Diseases Research Centre Institution Review Board (registration number: 00002911). The questionnaire contained an information sheet regarding the study and an informed consent statement for participants to agree to participate or not. All participants who declined to take part in the study were immediately withdrawn and could not proceed to respond to the questionnaire. Access to the online portal for the questionnaire was limited to investigators who were assigned user rights by the principal investigator to ensure confidentiality of collected data.

### Sample size determination

We used the methods for calculating the sample size for prevalence studies as described by Pourhoseingholi and others.^18^ Assuming an 82% prevalence of good knowledge and practice regarding COVID-19 obtained from a similar study among HCWs,^19^ a confidence level of 95% and a precision level of 5%, we obtained a minimum required sample size of 227 participants. By extrapolating to a finite population of 1900 medical laboratory personnel officially recognised under the medical laboratory register of the Biomedical Society of Zambia, we obtained a final sample size of 204 participants.

### Study design

This cross-sectional survey was conducted among 208 medical laboratory professionals in Zambia. Due to COVID-19 related restrictions imposed during this period, it was not feasible to conduct face-to-face interviews and therefore we administered an online questionnaire using Google Forms (Alphabet, Inc., Mountain View, California, United States).

### Data collection

Content validation of the questionnaire was done by administering the questionnaire to faculty in the Biomedical Sciences unit at the Copperbelt University, Ndola, Zambia. The results from the pre-test were not included in the final analysis but were used to modify the questionnaire based on feedback. The questionnaire had two main components: demographics and knowledge, attitudes and practices. The demographic section had 8 questions while the knowledge, attitudes and practices section was divided into the following subsections: knowledge (8 questions), attitude (5 questions) and practices (13 questions).

To ensure that only target respondents participated in the survey we distributed the link to the survey through the email database and WhatsApp (Facebook Inc, Menlo Park, California, United States) group facilitated by the Biomedical Society of Zambia during data collection from 10 to 29 June 2020. A total of 750 participants were availed the link for the questionnaire. There were 210 medical laboratory personnel who participated in the study, giving a response rate of 28%. Participants who consented to participate in the study by checking the consent box in the preliminary page of the questionnaire were allowed to proceed with the rest of the questionnaire. Only participants who completed the questionnaire by clicking on the ‘Submit’ button had their responses recorded.

### Data management and statistical analysis

Collected data were downloaded and cleaned in Microsoft Excel (Microsoft Corp., Redmond, Washington, United States) and exported to Statistical Package for Social Sciences version 23 (IBM Corp., Armonk, New York, United States) for statistical analysis. To ensure the internal consistency and reliability of the data, we used Cronbach’s alpha coefficient according to methods described in a previous study.^20^ We obtained an alpha value of 0.645, indicating adequate reliability.^21^ The outcome variables in this study were COVID-19 knowledge levels and practice towards COVID-19. Attitude was investigated but limited to descriptive analysis due to the limited number of questions. Eight questions were used to assess COVID-19 knowledge, each scoring 1 point for a correct answer. Thirteen questions were asked to determine whether a participant had good practice towards COVID-19 or not, each scoring 1 point for positive practice and 0 for negative practice. Bloom’s cut-off of 80%^22^ was used to determine good knowledge and good practice. Questions were adapted from a previous study.^23^

To identify factors that predict good knowledge and practice towards COVID-19, bivariate analysis was performed. All factors that were statistically significant as reported by 95% confidence interval (CI) in bivariate logistic regression were included in a forward stepwise multivariate logistic regression model to identify factors that were independently associated with good knowledge and practice.

## Results

### Demographic characteristics

A total of 208 medical laboratory professionals from seven provinces of Zambia took part in this study. There were more men (*n* = 121, 58.2%) than women. Half of the participants were aged 20–29 years while more than half had a diploma. Most of the respondents were from a hospital laboratory (*n* = 134, 64.4%) (Table 1).

**TABLE 1:** Demographic characteristics of medical laboratory professionals, Zambia, June 2020.

Factor	Number of participants (*n*)	%
**Sex**		
Female	87	41.8
Male	121	58.2
**Age (years)**		
20–29	104	50.0
30–39	79	38.0
≥ 40	25	12.0
**Current qualification**		
Certificate in Biomedical Sciences	2	1.0
Diploma in Biomedical Sciences	155	74.5
BSc Biomedical Sciences	40	19.2
MSc Biomedical Sciences	9	4.3
PhD	2	1.0
**Work experience**		
Less than 5 years	106	51.0
5–10 years	60	28.8
More than 10 years	42	20.2
**Facility type**		
Clinic laboratory	14	6.7
Health centre laboratory	26	12.5
Hospital laboratory	134	64.4
Private or non-governmental organisation	20	9.6
Others	14	6.7
**Participant’s province**		
Central	12	5.8
Copperbelt	61	29.3
Eastern	17	8.2
Lusaka	40	19.2
North-western	18	8.7
Northern	11	5.3
Southern	30	14.4
Others	19	9.1
**Total**	**208**	**100.0**

BSc, Bachelor of Science; MSc, Master of Science; PhD, Doctor of Philosophy.

Participants showed good knowledge regarding awareness of practical measures to stop the spread of COVID-19 (*n* = 208, 100.0%), the techniques used to test for COVID-19 (*n* = 208, 100.0%) and symptomatic management of COVID-19 (*n* = 202, 97.1%) (Table 2 and Table 3). One-quarter (*n* = 52, 25.0%) of the participants reported being involved in sampling for COVID-19 while slightly over a third (*n* = 73, 35.1%) reported having been trained in sample handing and transportation.

**TABLE 2:** Characteristics of responses for knowledge and practice towards COVID-19, Zambia, June 2020.†

KAP aspect	Question	Correct		Incorrect	
		*n*	%	*n*	%
Knowledge of COVID-19	The main clinical symptoms of COVID-19 are fever, fatigue, dry cough and myalgia	194	93.3	14	6.7
	COVID-19 is a disease caused by SARS-CoV-2	177	85.1	31	14.9
	The COVID-19 virus spreads via respiratory droplets of infected individuals	207	99.5	1	0.5
	Our institution has informed us about COVID-19	199	95.7	9	4.3
	I am aware of the practical measures to help stop the spread of COVID-19	208	100.0	0	0.0
	I am aware of the techniques used to test for COVID-19	208	100.0	0	0.0
	There currently is no effective cure for COVID-19, but early symptomatic and supportive treatment can be helpful	202	97.1	6	2.9
	Persons with COVID-19 cannot transmit the virus to others when a fever is not present	175	84.1	33	15.9

COVID-19, coronavirus disease 2019; KAP, Knowledge, Attitudes and Practices; SARS-CoV-2, severe acute respiratory coronavirus 2.

† , Number of participants = 208.

**TABLE 3:** Characteristics of responses for knowledge and practice towards COVID-19, Zambia, June 2020.†

KAP aspect	Question	Yes		No	
		*n*	%	*n*	%
Practices towards COVID-19	I practise social distancing during work	167	80.3	41	19.7
	I wear masks to help reduce transmission of COVID-19	199	95.7	9	4.3
	I put on PPE when processing samples	195	93.8	13	6.2
	I process samples from the respiratory tract in a safety cabinet	103	49.5	105	50.5
	I disinfect my work surfaces	204	98.1	4	1.9
	We effectively dispose of all processed samples	194	93.3	14	6.7
	We effectively dispose of disposable PPE	181	87.0	27	13.0
	I wear masks when going out	195	93.8	13	6.3
	I avoid crowded places	191	91.8	17	8.2
	I have been trained in laboratory safety against COVID-19	71	34.1	137	65.9
	I have been involved in sampling a COVID-19 suspected patient	52	25.0	156	75.0
	We have adequate sample referral systems	94	45.2	114	54.8
	I have been trained in packaging of COVID-19 samples	73	35.1	135	64.9

COVID-19, coronavirus disease 2019; KAP, Knowledge, Attitudes and Practices; PPE, personal protective equipment.

† , Number of participants = 208.

### Factors associated with COVID-19 knowledge

A high proportion of respondents were knowledgeable about COVID-19 (*n* = 175, 84.1%). Bivariate logistic regression analysis showed that having a bachelor’s degree compared to having a certificate or diploma (crude odds ratio (OR): 4.68, CI: 1.07–20.44) and COVID-19 training (crude OR: 8.72, CI: 2.02–37.65) among participants were associated with COVID-19 knowledge. Therefore, participants with higher academic qualifications and COVID-19 training were 4.68 and 8.72 times more likely to have good COVID-19 knowledge (Table 4).

**TABLE 4:** COVID-19 knowledge of participants and associated factors, Zambia, June 2020.

Factors	Knowledgeable about COVID-19				Crude odds ratio	95% confidence interval
	Yes	%	No	%		
**Sex**						
Male	104	86.0	17	14.0	1	-
Female	71	81.6	16	18.4	0.73	0.34-1.53
**Age (years)**						
20–29	88	84.6	16	15.4	1	-
30–39	65	82.3	14	17.7	0.84	0.39-1.85
≥ 40	22	88.0	3	12.0	1.33	0.36-4.99
**Current qualification**						
Certificate or diploma	126	80.30	31	19.7	1	-
Bachelor’s	38	95.0	-	25.0	4.68	1.07-20.44
Master’s or PhD	11	100.0	0	0.0	316182818.76	0.0
**Facility**						
Clinic and health centre laboratories	32	80.0	8	20.0	1	-
Hospital laboratories	111	82.8	23	17.2	1.21	0.49-2.95
Others†	32	94.1	2	5.9	4.00	0.79-20.32
**Participant’s province**						
Northern	8	72.7	3	27.3	1	-
Eastern	13	76.5	4	23.5	1.22	0.22-6.92
Others	15	78.9	4	21.1	1.40	0.25-7.90
Lusaka	32	80.0	8	20.0	1.50	0.32-6.97
North-western	15	83.3	3	16.7	1.88	0.31-11.52
Central	10	83.3	2	16.7	1.88	0.25-14.08
Southern	27	90.0	3	10.0	3.30	0.57-20.10
Copperbelt	55	90.2	6	9.8	3.44	0.71-16.55
**Trained in COVID-19**						
No	112	78.3	31	21.7	1	-
Yes	63	96.9	2	3.1	8.72	2.02-37.65
**Total**	**175**	**84.1**	**33**	**15.9**	**-**	**-**

COVID-19, coronavirus disease 2019; PhD, Doctor of Philosophy.

† , Private laboratories, research laboratories.

### Practice towards COVID-19 by medical laboratory personnel

Poor practices towards COVID-19 were recorded among three-quarters of the participants. Having a master’s degree or a Doctor of Philosophy compared to having a certificate or diploma (crude OR: 4.51, 95% CI: 1.30–15.70), private or research laboratories compared to clinic or health centre laboratories (crude OR: 3.09, 95% CI: 1.01–9.45) and COVID-19 training (crude OR: 12.97, 95% CI: 6.19–27.18) were associated with good COVID-19 practices (Table 5).

**TABLE 5:** COVID-19 practices of participants and associated factors, Zambia, June 2020.

Factors	Practice towards COVID-19				Crude odds ratio	95% confidence interval
	Poor	%	Good	%		
**Sex**						
Male	92	76.0	29	24.0	1	-
Female	64	73.6	23	26.4	1.14	0.61-2.15
**Age (years)**						
20–29	78	75.0	26	25.0	1	-
30–39	62	78.5	17	21.5	0.82	0.41-1.65
≥ 40	16	64.0	9	36.0	1.69	0.67-4.28
**Current qualification**						
Certificate or diploma	124	79.0	33	21.0	1	-
Bachelor’s	27	67.5	13	32.5	1.81	0.84-3.89
Master’s or PhD	5	45.5	6	54.5	4.51	1.30-15.70
**Facility**						
Clinic and health centre laboratories	34	85.0	6	15	1	-
Hospital laboratories	100	74.6	34	25.4	1.93	0.74-4.99
Others†	22	64.7	12	35.3	3.09	1.01-9.45
**Participant’s province**						
Central	11	91.7	1	8.3	1	-
Northern	10	90.9	1	9.1	1.10	0.06-20.01
North-western	16	88.9	2	11.1	1.38	0.11-17.09
Southern	25	83.3	5	16.7	2.20	0.23-21.11
Others‡	15	78.9	4	21.1	2.93	0.29-30.01
Copperbelt	46	75.4	15	24.6	3.59	0.43-30.14
Eastern	10	58.8	7	41.2	7.70	0.80-74.05
Lusaka	23	57.5	17	42.5	8.13	0.96-69.17
**Trained in COVID-19**						
No	129	90.2	14	9.8	1	-
Yes	27	41.5	38	58.5	12.97	6.19-27.18
**Total**	**156**	**75**	**52**	**25**	**-**	**-**

COVID-19, coronavirus disease 2019, PhD, Doctor of Philosophy.

† , Private laboratories, research laboratories

‡ , Luapula, Muchinga and Western provinces

### Attitude towards COVID-19 among medical laboratory personnel

About 93.8% of participants reported that they would accept isolation from the community if diagnosed with COVID-19. A few (46.6%) would accept to be vaccinated against COVID-19 if a vaccine was available. On the other hand, many participants (97.6%) were ready to take part in anti-epidemic community activities. Our study revealed that 77.9% were confident that Zambia could win the battle against the COVID-19 virus (Table 6).

**TABLE 6:** Attitude of biomedical professionals towards coronavirus disease 2019, Zambia, June 2020.

Variables	Response	
	*n*	%
**Would accept isolation from the community if diagnosed with COVID-19**		
Yes	195	93.8
No	8	3.8
Don’t know	5	2.4
**Ready to participate in anti-epidemic activities in the community**		
Yes	203	97.6
No	2	1.0
Don’t know	3	1.4
**Agree that COVID-19 will finally be successfully controlled**		
Yes	183	88.0
No	10	4.8
Don’t know	15	7.2
**Would accept to be vaccinated against COVID-19 if a vaccine was available**		
Yes	97	46.6
No	75	36.1
I don’t know	36	17.3
**Have confidence that Zambia can win the battle against the COVID-19 virus**		
Yes	162	77.9
No	24	11.5
Don’t know	22	10.6
**Total**	**208**	**100.0**

COVID-19, coronavirus disease 2019.

### Factors independently associated with good knowledge and practice about COVID-19

After controlling for possible confounding factors, having a higher current qualification (bachelor’s degree) and COVID-19 training were independently associated with good COVID-19 knowledge among biomedical professionals in Zambia. Similarly, having a higher current qualification (master’s degree or a Doctor of Philosophy) and COVID-19 training were independently associated with good COVID-19 practice (Table 7).

**TABLE 7:** Factors independently associated with good COVID-19 knowledge and practice, Zambia, June 2020.

Independent factors	Adjusted odds ratio	95% confidence interval
**Good COVID-19 knowledge**		
**Current qualification**		
Certificate or diploma	1	-
Bachelor’s	5	1.13–22.19
Master’s or PhD	316182818.8	0
**Trained in COVID-19**		
No	1	-
Yes	8.83	2.03–38.44
**Good COVID-19 practices**		
**Current qualification**		
Certificate or diploma	1	-
Bachelor’s	2.35	0.93–5.95
Master’s or PhD	5.23	1.15–23.87
**Trained in COVID-19**	-	-
No	1	-
Yes	14.01	6.47–30.36

COVID-19, coronavirus disease 2019, PhD, Doctor of Philosophy.

## Discussion

Very few studies worldwide have documented knowledge, attitudes and practices among HCWs towards COVID-19 due to the novel nature of the disease.^22^ Our study findings showed that the majority of medical laboratory professionals in Zambia had good knowledge of COVID-19. The level of knowledge on COVID-19 among participants was similar irrespective of their gender, age and laboratory facility. These findings are encouraging as they indicate that there are no inherent differences in knowledge of COVID-19 among groups based on unique demographic characteristics in the population. The majority of participants exhibited poor practices towards COVID-19 contrary to findings from Uganda and Nepal.^19,22^ Differences in knowledge and practice regarding COVID-19 have been reported before in a study by Asemahagn.^24^ Most participants with poor practices were those who had certificate qualifications, those without prior COVID-19 training and those from clinic and health centre laboratories. This could be attributed to limited resources, health information and laboratory materials in most clinic and health centre laboratories found in rural areas. Poor practices can lead to delayed or wrong laboratory diagnosis, leading to poor patient management or safety incidents that could harm the personnel and their immediate co-workers, families and patients or laboratory clients.^25^

Current qualification and COVID-19 training among participants were significantly associated with good COVID-19 knowledge, a finding similar to that obtained by a study in Vietnam,^26^ but contrary to the findings of Bhagavathula and others.^27^ Our findings show that participants with higher academic qualifications and COVID-19 training were 4.68 and 8.72 times more likely to have good COVID-19 knowledge. The current qualification was also significantly associated with good COVID-19 practices which agrees with study findings from Uganda.^22^ On the other hand, the type of laboratory facility and COVID-19 training were significantly associated with good COVID-19 practices. This shows that participants who received COVID-19 training were 12.97 times more likely to have good practices towards COVID-19 and general infection prevention; this is in agreement with similar studies in Nepal and Ethiopia.^19,28^

### Limitations

The study may be susceptible to self-presentation bias as it was based on an online questionnaire. The study was limited by the level of responses available and could have been strengthened by increasing the range of answers using, for example, a five-point Likert scale. The questions on attitude were limited and therefore not powerful enough to generate meaningful conclusions on the attitude of respondents. The study could not capture dropouts and respondents who refused consent as only individuals who consented and submitted the questionnaire had their responses recorded.

### Conclusion

Our study found that medical laboratory professionals in Zambia have good knowledge regarding COVID-19. The current qualification and COVID-19 training were independently associated with COVID-19 knowledge and practice. As cases of COVID-19 continue to be recorded in the country, there is need for continuous professional development among medical laboratory personnel as a key intervention in improving their contribution to COVID-19 control efforts.
